# Friend or Foe? The Roles of Antioxidants in Acute Lung Injury

**DOI:** 10.3390/antiox10121956

**Published:** 2021-12-07

**Authors:** Yang Liu, Shujun Zhou, Du Xiang, Lingao Ju, Dexin Shen, Xinghuan Wang, Yanfeng Wang

**Affiliations:** 1 Department of Urology, Zhongnan Hospital of Wuhan University, Wuhan 430071, China; yliu2015@whu.edu.cn (Y.L.); 2018203030059@whu.edu.cn (D.S.); 2 Zhongnan Hospital of Wuhan University, Institute of Hepatobiliary Diseases of Wuhan University, Transplant Center of Wuhan University, Wuhan 430071, China; shujunzhou@whu.edu.cn (S.Z.); xiangdd@whu.edu.cn (D.X.); 3 Department of Biological Repositories, Zhongnan Hospital of Wuhan University, Wuhan 430071, China; julingao1990@whu.edu.cn; 4 Human Genetics Resource Preservation Center of Hubei Province, Wuhan 430071, China

**Keywords:** acute lung injury, reactive oxygen species, oxygen radical, antioxidants

## Abstract

Acute lung injury (ALI) is an acute hypoxic respiratory insufficiency caused by various intra- and extra-pulmonary injury factors. The oxidative stress caused by excessive reactive oxygen species (ROS) produced in the lungs plays an important role in the pathogenesis of ALI. ROS is a “double-edged sword”, which is widely involved in signal transduction and the life process of cells at a physiological concentration. However, excessive ROS can cause mitochondrial oxidative stress, leading to the occurrence of various diseases. It is well-known that antioxidants can alleviate ALI by scavenging ROS. Nevertheless, more and more studies found that antioxidants have no significant effect on severe organ injury, and may even aggravate organ injury and reduce the survival rate of patients. Our study introduces the application of antioxidants in ALI, and explore the mechanisms of antioxidants failure in various diseases including it.

## 1. Introduction

Acute lung injury (ALI) is defined as diffuse pulmonary interstitial edema, alveolar edema and an acute hypoxic respiratory insufficiency, caused by damage to alveolar epithelial cells and capillary endothelial cells [1]. ALI is caused by a variety of non-cardiogenic factors such as severe infection, shock and burns [1]. The clinical manifestations of ALI are progressive hypoxemia or acute respiratory distress syndrome (ARDS) [2]. ARDS is a syndrome characterized by tachypnea, hypoxemia, loss of pulmonary compliance, and diffuse alveolar infiltration [3]. The severity of ALI/ARDS is associated with a poor prognosis and high mortality. Although considerable progress has been made in the study of the pathogenesis and molecular mechanisms of ALI/ARDS, its morbidity and mortality remain high [4]. Due to the heterogeneity of the etiology and the complexity of complications of ALI/ARDS, the current mode of treatment of ALI/ARDS is mainly supportive treatment, which focuses on the treatment of underlying diseases and bedside care, including mechanical ventilation and the use of corticosteroids [5]. As a standard treatment to support lung oxygenation, mechanical pulmonary ventilation can also cause physical damage to the lungs during ventilation, aggravating lung inflammation and the clinical symptoms of patients [6]. The symptoms of ALI/ARDS can also be relieved to a certain extent through the use of glucocorticoids [7]. Ashbaugh et al. [2] confirmed that ALI/ARDS patients with fat embolism, cyanosis, and lethargy were significantly improved after 5 days of intravenous glucocorticoid injection, while other patients did not benefit from the use of glucocorticoids.

The pathogenesis of ALI/ARDS is very complicated. A variety of factors stimulate the accumulation and activation of inflammatory cells in the lung, and inflammatory cells release pro-inflammatory factors such as reactive oxygen species (ROS), tumor necrosis factor-alpha (TNF-α), interleukins (ILs) and elastase, which promote the inflammatory cascade [8,9]. Oxidative stress is one of the major causes of pulmonary vascular endothelial cell damage and alveolar epithelial cell dysfunction. ROS can destroy pulmonary microvascular endothelial cells and epithelial cells in different ways, which increases pulmonary vascular permeability and promotes the formation of a pulmonary edema [10]. In addition, ROS can also interact with a variety of cytokines to promote the expression of pro-inflammatory cytokines and adhesion molecules such as ICAM-1/VCAM-1, thereby promoting the formation of the inflammatory cascade [11]. Oxidation-antioxidant balance is critical to vascular homeostasis, and antioxidants have gradually become one of the important methods for the treatment of ALI [12,13,14]. However, the effectiveness of antioxidants against various diseases has been questioned in recent years. We have summarized the mechanisms of ROS production in ALI and antioxidant treatments for ALI, and explored the possible mechanisms of antioxidant failure in various diseases including ALI.

## 2. The Role of ROS in ALI

### 2.1. ROS Generation

**Figure 1:**
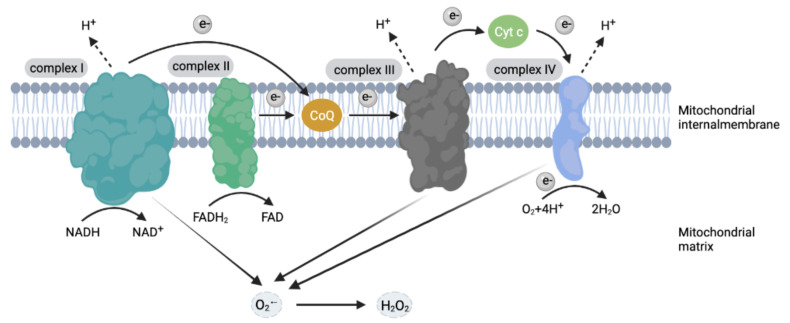
Sources of reactive oxygen species. NADH: nicotinamide adenine dinucleotide; NAD^+^: nicotinamide adenine dinucleotide; FADH_2_: flavine adenine dinucleotide, reduced; FAD: flavine adenine dinucleotide; Cyt c: cytochrome C; CoQ: coenzyme Q.

ROS includes oxygen radicals, such as superoxide anion radicals (O_2_^•−^) and hydroxyl radicals (^•^OH) and non-radical oxidants, such as hydrogen peroxide (H_2_O_2_) and singlet oxygen (O_2_) [15]. Various types of cells in the lung can produce ROS, including endothelial cells, neutrophils, eosinophils, alveolar macrophages and alveolar epithelial cells [16]. In addition, fibroblasts, perivascular fat cells and vascular smooth muscle cells are also the important sources of ROS in the vascular system [17]. The electron transport chain in mitochondria is the main source of ROS. Mitochondria perform a series of single-electron transfers through electron transfer complexes (ETC), such as complex I (reduced form of nicotinamide-adenine dinucleotid/NADH dehydrogenase), complex III (cytochrome c oxidase) and complex IV (cytochrome c reductase) [18]. One to two percent of the electrons leak during the conversion of ADP to ATP and react with O_2_ to form O_2_^•−^ [17]. Mitochondrial dysfunction and the interruption of the electron transport chain in lung cells during ALI leads to the excessive production of ROS (Figure 1). The plasma membrane and endoplasmic reticulum of lung endothelial cells are rich in Nicotinamide adenine dinucleotide phosphate oxidase (NOXs), which is one of the important sources of ROS [19]. Jin et al. [20] showed that the use of NOXs inhibitor-apocynin could significantly reduce the expression of NOX2 and NOX4_,_ and further reduce the production of ROS, while inhibiting the inflammatory response induced by the NF-kB pathway. Consequently, the use of NOXs inhibitor-apocynin alleviates ALI effectively induced by acute pancreatitis in rats. Jiang J et al. [21] confirmed that NOX4 knockdown attenuated the redox-sensitive activation of the *CaMKII/ERK1/2/MLCK* signal pathway, and restored the expression of tight junction proteins ZO-1 and occludin in lung endothelial cells to maintain the integrity of the barrier of endothelial cells, which suggested that targeting NOX4 is an innovative and effective treatment for ALI. In the vascular system of the lungs, endothelial nitric oxide synthase (eNOS) and xanthine oxidase (XO) are also the main sources of ROS [22]. Under the catalysis of eNOS, NO combines with O_2_^•−^ to form peroxynitrite ONOO^−^, which is a molecule with strong oxidizing ability [22]. ONOO^−^ promotes cell death by interfering with many cell physiological processes, and produces many cytotoxic substances, such as ^•^OH, nitrogen dioxide and carbonate free radicals [22]. Xanthine oxidoreductase (XOR), composed of two different forms including xanthine dehydrogenase (XDH) and XO, is involved in the purine metabolism and the production of ROS in the human body [23]. Both XDH and XO can use xanthine and O_2_ as substrates to generate H_2_O_2_ and O_2_^•−^ [24].

### 2.2. The Oxidation Function of ROS

ROS, as a new signal mediator involved in cell growth, differentiation, progression, and death, has attracted extensive attention, and participates in ALI in a variety of ways. To begin with, the inflammatory cells can release a large amount of ROS in the lungs, which eliminates pathogens effectively. However, during tissue repair in the late stages of inflammation, the accumulation of excessive ROS produced by inflammatory cells causes irreversible damage to the DNA of new cells, including point mutations, deletions, and rearrangements of DNA [25]. In addition, the generation of excessive oxygen free radicals inside and outside the cell triggers a variety of destructive reactions. Oxygen free radicals can combine with the lipids on the plasma membrane surface to cause lipid peroxidation to form new free radicals, which are extremely unstable and easily decomposed into carbonyl groups, malondialdehyde and volatile hydrocarbons, such as ethane and pentane [26,27]. The process of lipid peroxidation destroys the structure of cell membranes, resulting in changes to the physical properties of the phospholipid bilayer. Another relevant point to make is that many types of proteins are directly oxidized by ROS and lose their original structure and function [28].

### 2.3. ROS Induces Calcium Channel Dysfunction

**Figure 2:**
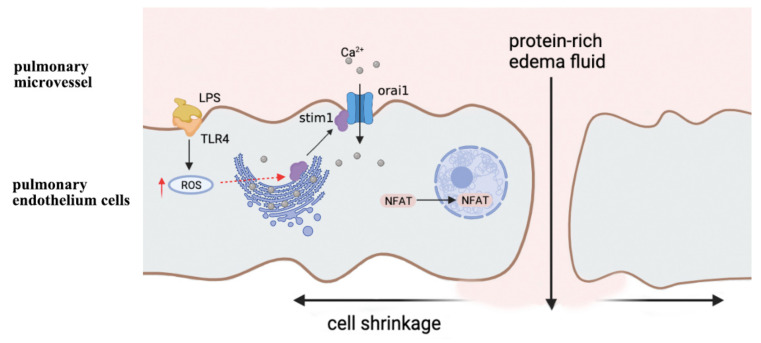
Calcium oscillations and endothelial dysfunction during acute lung injury. LPS: endotoxins; TLR4: toll-like receptor 4; ROS: reactive oxygen species; stim1: stromal interaction molecule 1; Orai1: calcium release-activated calcium modulator 1; NFAT: nuclear factor of activated T cells.

The concentration of calcium ions rapidly changes when cells are subjected to physiological stimulation. The increase in the calcium ion concentration usually occurs in the form of “calcium oscillation”, that is, calcium ion concentration forms multiple calcium peaks over time, when calcium ions enter the cytoplasm according to certain rules [29]. Intracellular calcium oscillations are usually regulated in two ways, including frequency regulation (FM) and amplitude regulation (AM). The occurrence of calcium oscillations in alveolar endothelial cells is considered to be one of the key factors that lead to endothelial cell damage and inflammation [30]. When the body is infected, blood-derived endotoxins (LPS) combine with Toll-like receptors 4 (TLR4) on alveolar endothelial cells, leading to an increase in the level of ROS in the cytoplasm [31]. Subsequently, ROS can induce s-glutathione in the cysteine residues of stromal interaction molecule 1 (Stim1) located on the endoplasmic reticulum. Stim1, as a sensor of oxidative stress, activates store-operated calcium entry (SOCE) to generate calcium oscillations [31]. During the progression of ALI, the accumulation of ROS causes Stim1 in the endoplasmic reticulum of alveolar endothelial cells to migrate and interact with calcium channel protein-Orai1 on the cell membrane, leading to the occurrence of calcium oscillation, which causes the shrinkage of the alveolar endothelial cell membrane and the flow of protein-rich edema fluid from the intercellular space into the alveolar cavity [31]. In addition, the activation of Stim1 can regulate the nuclear translocation of nuclear factors of activated T cells (NFAT). A previous study showed that the calcium-mediated NFAT signal might act as a key signal for the pathological contraction of alveolar endothelial cells [32] (Figure 2). The use of Orai1 channel inhibitor BTP2 can significantly inhibit the nuclear translocation of NFAT, which reduces endothelial cell apoptosis and alveolar edema, and improving the lung ventilation capacity [31].

In addition to the activation of NFAT and Orai1 by TLR4-induced ROS, a large number of genes and their downstream expressions were also changed. A previous study found that Btk/PLCγ/IP3R signaling pathway plays an important role in ALI [33]. The level of calcium ions and the apoptosis rate in human umbilical vein endothelial cells decreased significantly with the silencing of *Btk* or *PLCγ* genes [33]. Similarly, the inflammatory cell infiltration in lung tissue expressively reduced while the survival rate of mice also pointedly increased in ALI mice [33]. TRPC6 is a classic transmembrane protein channel that has an important regulatory effect on calcium ions. A previous study reported that TRPC6 induces a calcium influx in endothelial cells [34]. ROS-mediated activation of TRPC6 calcium channels is a key factor in pulmonary vascular leakage and inflammation [35].

### 2.4. ROS Induces Chemotaxis of Neutrophils

The infiltration of neutrophils in the alveolar and pulmonary interstitial is a typical feature of ALI. Neutrophils release a variety of active substances, including ROS, antimicrobial peptides, neutrophil extracellular traps (NETs), and many pattern recognition receptors, which provide an effective means of removing pathogens and the key factor of aggravating local inflammation [36]. A previous study showed that the mortality caused by ALI is closely related to the degree of neutrophil infiltration [37]. NOXs in phagocytes release a large amount of ROS, namely “respiratory burst” or “oxidative burst” during ALI [38,39]. As a chemotactic agent for neutrophils, excessive ROS recruits circulate neutrophils to infiltrate the lungs [40]. Hattori H et al. [41] confirmed that ROS could promote the migration of neutrophils to alveoli by driving the glutathionylation of actin. The regulation of actin dynamics is critical for cell migration and adhesion, and high concentrations of ROS can promote neutrophil migration by promoting glutathione of neutrophils and increasing the expression of actin and related proteins [41]. Among the many superoxides, H_2_O_2_ has the highest membrane permeability and is an important initiating factor for neutrophil glutathionylation [42].

Moreover, the voltage-gated proton channel Hv1/VSOP can counteract the charge imbalance in the cell through proton transportation to reduce the generation of ROS in the cell, which reduces the migration ability of neutrophils [43]. Okochi et al. [43] found that when neutrophils with blocked Hv1/VSOP channels were stimulated, ROS production increased by 2.5 times, which resulted in a significant increase in the migration ability of neutrophils. In addition, the triggering receptor expressed on myeloid cells (TREM-1) plays an important role in the regulation of inflammatory signals and the migration of neutrophils into the alveoli. A study showed that inhibiting the expressions of NOX2 or TREM-1 alone decreases the chemotaxis of neutrophils significantly [44]. Additionally, the effect of TREM-1 on the chemotaxis of neutrophils is dependent on the superoxide produced by NOX2 [44].

### 2.5. Cell-Free Hemoglobin (CFH) and ALI

Intra-alveolar hemorrhage is a common clinical symptom of ALI/ARDS. In ARDS, the oxidative environment in the alveoli and the lack of hemoglobin-processing proteins create an ideal environment for red blood cells to pass through lung endothelial cells and epithelial cells to cause damage during ALI [2,45]. A previous study demonstrated that the perfusion of red blood cells in the airways of rats can cause ALI, and the mechanism may be closely related to CFH which is broken down after the rupture of red blood cells [46]. CFH is derived from the breakdown of hemoglobin, myoglobin, horseradish peroxidase, cytochrome B5 and cytochrome P450, which can cause oxidative damage and impair cell integrity [47]. Shaver et al. [48] found that CFH in the alveoli disrupted the alveolar-capillary barrier. CFH in the alveoli is a rich source of redox active iron. It can destroy lipids, proteins and DNA in alveolar epithelial cells and weaken the cells’ ability to clear pulmonary edema fluid [49]. In addition, Aggarwal et al. [50] reported that the application of hemopexin to ARDS model mice can improve lung epithelial Na^+^ channel (ENaC) activity, reduce pulmonary edema fluid retention, and improve lung function.

## 3. The Application of Antioxidants in ALI

### 3.1. Acetylcysteine (NAC)

The reduced glutathione is a key substance in maintaining redox homeostasis. However, due to poor permeability, the exogenous administration of glutathione is often ineffective [51]. NAC, the precursor of glutathione, can easily penetrate cell membranes and then deacetylate to form l-cysteine to participate in the synthesis of glutathione [52]. NAC has a significant effect on ALI. A study using NAC in the treatment of ALI in pigs demonstrated that NAC treatment could maintain the integrity of the tracheal epithelial structure and reduce the degree of the epithelial edema and inflammatory infiltration [53]. Likewise, the application of NAC can improve the oxygenation of the rabbit with ALI significantly, reduce pulmonary edema and airway hyperresponsiveness [54], whereas the results of the clinical trials of NAC in the treatment of ALI/ARDS are confusing. Previous studies demonstrated that the application of NAC could effectively reduce ALI in patients [55,56,57,58]. However, a study that included 5 randomized clinical trials with 183 patients, reported that the use of NAC did not reduce the short-term mortality of ALI patients significantly [59]. Interestingly, intravenous NAC has been proven to play a key role in the treatment of severe cases of COVID-19 [60,61,62]. NAC can treat ALI by antagonizing cell peroxidation damage caused by various reasons. NAC can upregulate the nuclear transcription factor Nrf2 and inhibit the expression of NF-kB to scavenge oxygen free radicals, thereby reducing inflammation [63,64,65]. Correspondingly, NAC also plays an important role in alleviating cytokine storms. NAC can effectively regulate the expression of cytokines such as Th1, Th2, and Th17, while the combination of NAC and Dex can achieve better efficacy [66].

### 3.2. Vitamins

Vitamins, as a common type of antioxidant, have been reported to produce a protective effect in ALI. A previous study confirmed that the intermittent administration of vitamin A from the airway could effectively reduce inflammation in the lungs of ALI rats and promote the maturation of new alveoli [67]. Vitamin B2 (riboflavin) is a common antioxidant. After the treatment of ALI in rats with vitamin B2, the level of malondialdehyde (MDA) and the activity of myeloperoxidase (MPO) in rats were significantly reduced, and the effect of vitamin B2 was dose-dependent in a certain range [68]. Vitamin E is a lipophilic antioxidant. Sufficient amounts of alveolar surfactants are essential for maintaining normal lung function. About 90% of surfactants are lipids, including dipalmitoyl-phosphatidylcholine, cholesterol, polyunsaturated lipids and other important functional molecules [69,70,71]. Because these lipids are extremely sensitive to oxidation, the lipophilic antioxidant vitamin E plays a valuable role in the antioxidant protection system of the lungs. Previous studies showed that the concentration of vitamin E in the lungs increases significantly in ALI, which is caused by various extreme conditions, possibly due to the body’s defense mechanisms [72,73]. In addition, vitamin E deficiency can cause more severe lung histological damage in rats [74]. However, several clinical studies showed that the administration of vitamin E (or vitamin E acetate) had not achieved the expected efficacy [75].

### 3.3. Ambroxol

Ambroxol, a mucolytic drug, is used in various respiratory diseases such as acute and chronic bronchitis and bronchial asthma, bronchiectasis, emphysema, tuberculosis, and pneumoconiosis. In the treatment of ALI, ambroxol mainly plays the role of an antioxidant, inhibiting inflammation and mucus secretion [76]. More than twenty years ago, ambroxol was proven to effectively improve paraquat-induced ALI and reduce lipid peroxidation caused by H_2_O_2_ [77]. Ge LT et al. [78] found that ambroxol can inhibit the expression of cell mucin (MUC5AC) and phosphorylated-extracellular regulated protein kinases (p-Erk), thereby reducing edema and inflammation in the lung. Interestingly, they also found that the administration of ambroxol via airway inhalation was concentration-dependent in mitigating the effects of inflammatory cytokine intervention [78]. Unlike NAC, which acts as a precursor of direct antioxidants, the antioxidant effect of ambroxol is achieved indirectly. Ambroxol can reduce the recruitment of various immune cells in the lungs effectively, and reduce the secretion of cytokines such as TNF-α, IL-6, and TGF-β1, thereby reducing local inflammation and the level of oxidative stress [79].

### 3.4. Natural Medicine

Natural medicines are mainly derived from plants, animals, marine organisms and minerals. A variety of natural medicines that are derived from plant extracts have been shown to have significant effects on ALI. Resveratrol is a non-flavonoid polyphenol organic compound [80]. Jiang L et al [81]. showed that the pretreatment of resveratrol could effectively inhibit the activation of NLRP3 inflammasomes and reduce the level of IL-18 and IL-1β in ALI mice, which reduces the inflammatory infiltration of the lungs. Yang L et al. [82] also confirmed that resveratrol can reduce inflammation infiltration and inhibit cell apoptosis. Curcumin is a compound extracted from the rhizomes of plants in the Zingiberaceae and Araceae family [83]. The therapeutic effect of curcumin on ALI has been widely reported on. A previous study showed that curcumin might inhibit the activation of NLRP3 inflammasome-dependent pyrolysis by upregulating SIRT1, which in so doing inhibits inflammation [84]. Moreover, a large number of studies showed that curcumin has a significant inhibitory effect on IL-17A-mediated downstream pathways, including MCM2, MCM3, MCM6 [85], SMAD-dependent pathway *(SMAD2/SMAD3*) and non-SMAD-dependent (*JAK1/JAK2, STAT-1/STAT-3*) pathway [86], *p53/PAI-1* signaling pathway [87]. Interestingly, to improve the anti-inflammatory effect of curcumin, it is often loaded on a carrier and delivered to the lungs. Kim et al. [88] used cholesterol-coupled polyamides to deliver curcumin and heme oxygenase (HO-1) to achieve anti-inflammatory effects in the lungs of ALI mice via the trachea. A recent study delivered curcumin to the lungs of ALI mice through exosomes [89]. In addition, a previous study reported the effects of various natural plant extracts in the treatment of ALI. These natural medicines are primarily used to inhibit inflammation and reduce oxidative stress in lung tissue. These natural medicines include water extract of Taraxacum mongolicum hand-Mazz [90], stem bark of paulownia tomentosa steud [91], Naringenin [92], Emodin [93], Xanthohumol [94] and scutellaria extract scutellarin [95].

### 3.5. Micronutrients

Some micronutrients including zinc (Zn) and selenium (Se) have antioxidant effects and participate in the synthesis of various antioxidant enzymes, such as ceruloplasmin, superoxide dismutase (SOD), and glutathione peroxidase (GSH-Px). Zn is an essential micronutrient for the human body, and its antioxidant effect can protect the human body from an attack of ROS [96]. It can promote the formation of disulfide bonds of proteins to prevent the protein from being oxidized, thereby maintaining the integrity of the biofilm. Metallothionein (MT) can scavenge ROS and exert its antioxidant effect, and its transcription is strengthened by metal-regulatory transcription factor (MTF-1), which requires Zn in its formation [97,98]. Meanwhile, the activity of SOD was reported to be positively correlated with Zn levels between the plasma and liver [99]. Zn can also increase the accumulation of GSH in cells, and exert its antioxidant effect by synthesizing alanine and GSH, thereby further regulating cell metabolism and redox balance [100]. Zinc deficiency enhances hyperoxygen lung injury [101,102], while exogenous zinc effectively ameliorates acute lung injury induced by hyperoxygen [103] or carbon tetrachloride [104]. In addition, Wessels I et al. [105] confirmed that zinc pretreatment can significantly reduce the recruitment of neutrophils in the lung and inhibit the overactivity of neutrophils, thereby reducing ALI.

Se is an essential component of GSH-Px. The presence of Se can increase activity of GSH-Px, promote the decomposition of lipid peroxides, enhance the repair of sulfur compound damage that is caused by ROS, and maintain the integrity of cell membranes. However, only low concentrations of Se can scavenge ROS to exert its antioxidant effect, while high concentrations of Se can catalyze the generation of ROS. Previous studies have confirmed that selenoproteins play important roles in the regulation of the redox state, antioxidant defense and the production of pro-inflammatory cytokines [106,107]. Kim KS et al. [108] found that selenium post-treatment could activate GPx and reduce lipid peroxidation and ALI in the early stages following paraquat poisoning. Furthermore, the combined treatment of niacin and selenium reduced ALI and improved survival rates during sepsis. The mechanism of this combination therapy relates to the synergistic activation of the glutathione redox cycle, a decrease in the hydrogen peroxide level, and the upregulation of nuclear factor red blood cell 2 related factor 2 [109].

## 4. The Possible Mechanisms of Antioxidants Failure in Severe Organ Injury

In 1956, Harman D et al [110]. proposed the “free radical theory” and discovered that the pathogenesis of radiation-induced tumors is related to free radicals. In 1969, McCord JM et al. [111] reported the biological effects of SOD in anti-oxidation, which presents new avenues for free radical biology. Since then, a large number of studies have found that free radicals not only promote aging but are also related to the occurrence of various diseases, such as ALI, cancers, cardiovascular and cerebrovascular diseases, neurodegenerative diseases, arthritis and diabetes [112,113,114,115,116,117,118]. Therefore, antioxidants presented great potential in the prevention and treatment of various diseases including ALI.

However, in recent years, more and more studies have cast doubt on the free radical theory and the anti-oxidation theory of disease prevention. A randomized, double-blind, placebo-controlled, multicenter clinical trial by Rice TW et al. enrolled 272 adults with ALI requiring mechanical ventilation within 48 h [119]. Among them, 143 participants were supplemented with omega-3 fatty acids, gamma-linolenic acid and antioxidants twice a day. This clinical trial confirmed that supplementation with omega-3 fatty acids, γ-linolenic acid and antioxidants did not improve the clinical outcomes and the lung physiological function of patients with ALI, and cannot reduce the number of systemic inflammation markers and may even be harmful [119].

Sometimes, antioxidants are ineffective against certain diseases other than ALI. Some studies suggest that antioxidant therapy does not reduce mortality, and may even increase it. The Heart Protection Study Collaborative Group in the United Kingdom conducted a randomized clinical trial that included 20,536 British adults with coronary heart disease, other occlusive artery diseases or diabetes who were randomly assigned to receive antioxidant vitamin supplements (600 mg of vitamin E, 250 mg of vitamin C, and 20 mg of β-carotene daily) or a matching placebo. After receiving these antioxidant vitamin supplements, the participants’ plasma levels of α-tocopherol roughly doubled, vitamin C levels increased by a third, and β-carotene levels quadrupled. Interestingly, although the regimen significantly increased blood vitamin concentrations, it did not significantly reduce the incidence of any vascular disease, cancer, or 5-year mortality in patients [120].

Furthermore, Bjelakovic G et al. [121] conducted a systematic review including 78 primary and secondary randomized clinical trials. The study included 296,707 participants, consisting of 215,900 healthy participants and 80,807 patients with various diseases. All antioxidants were administered only orally, or in combination with vitamins and minerals. The duration of antioxidant supplementation ranges from 28 days to 12 years and the results of this study showed that β-carotene, vitamin E and high doses of vitamin A seem to increase mortality [121].

### 4.1. Possible Mechanism 1: The Dosage and Toxicity of Antioxidants

Antioxidant supplementation is necessary for certain patients. However, an excessive supplementation of antioxidants does not bring additional benefits and may even lead to potential toxicity. Miller ER et al. [122] conducted a meta-analysis. including 135,967 participants in 19 clinical trials. The dose of vitamin E taken by the participants in the study ranged from 16.5 to 2000 IU/d (median, 400 IU/d). The study confirmed that a statistically significant relationship exists between the vitamin E dose and the all-cause mortality of participants. The mortality risk increased when participants took vitamin E doses greater than 150 IU/ day, and high doses of vitamin E (≥400 IU/ day) supplements may increase all-cause mortality [122].

Vitamin E is considered to be a relatively safe type of vitamin [123], but long-term high-dose vitamin E may cause various adverse reactions [124]. A high dose of vitamin E exerts a pro-oxidation effect in vivo, and the pro-oxidation effect of vitamin E on LDL relates to the production of α-tocopherol free radical [125,126]. In addition, high doses of vitamin E may replace other fat-soluble antioxidants, disrupt the natural balance of the antioxidant system, and increase the vulnerability of oxidative damage [127]. Similarly, vitamin C acts as a powerful antioxidant that protects cells from oxidative damage by inhibiting the production of free radicals. However, the high-dose of vitamin C exerts cytotoxicity by producing excessive ROS and blocking energy homeostasis [128]. In general, the double-sided character of some antioxidants has been widely confirmed.

### 4.2. Possible Mechanism 2: Normal Physiological Function of Free Radicals and Hormesis Theory

It has been proven that the excessive generation of oxygen free radicals has a series of negative effects on the body, but it should not be ignored that free radicals, as normal metabolites of the human body, are also beneficial for maintaining the normal metabolism of the body. Free radicals actively participate in the anti-infective function of the immune system. In the process of phagocytosis of bacteria by immune cells, the oxygen consumption of immune cells increases sharply, producing a large amount of O^2−^ and H_2_O_2_, which will further produce ^•^OH through the Haber–Weiss reaction [129,130]. The above-mentioned active oxygen has a strong bactericidal effect. Immune cells use free radicals as a weapon to eliminate pathogenic microorganisms. Severe infection is one of the important causes of ALI, so the excessive use of antioxidants to reduce the level of oxygen free radicals may be harmful.

In contrast to the free radical harm theory, the Hormesis theory advocated by Calabrese EJ and Baldwin L asserts that various low-intensity harmful stimuli will not cause obvious damage to the body, but can induce the body to actively adjust its physiological activities and activate and strengthen the body’s defense mechanism [131,132]. Under normal circumstances, the balance between oxidative stress levels and antioxidant levels in the human body is stable, and a slight increase in the oxidative stress level does not exceed the antioxidant load. Slightly elevated free radicals are eliminated by the body’s defense mechanisms rather than external antioxidants. The abuse of antioxidants destroys the original oxidation-antioxidant balance to a certain extent.

## 5. Conclusions

Antioxidants once held great promise as a cure for various diseases, but a large number of clinical studies showed that antioxidants could have detrimental effects including increased mortality. In brief, the biological activity of antioxidants is similar to a two-sided coin, and its mechanism has yet to be studied. In addition, combination therapy with different targets may contribute to improving the efficacy of antioxidants. A continuous positive airway pressure (CPAP) ventilator can significantly improve the respiratory function, maintain positive airway pressure in patients, stabilize the contour of the chest, expand the alveoli and increase the functional residual capacity to prevent alveolar collapse. In addition, CPAP ventilator can reduce airway resistance, reduce intrapulmonary shunt while increasing oxygenation indicators, and reduce the consumption of lung surfactants [131]. However, a CPAP ventilator alone cannot cure ALI/ARDS, although, combined with ambroxol, it can significantly improve on its therapeutic effects [133]. Similarly, the previous study demonstrated that ambroxol, combined with pulmonary surfactant, has a better clinical effect in the treatment of ALI/ARDS, which can effectively improve the patient’s oxygenation index, reduce the time of oxygen therapy and the time of assisted ventilation [134]. In conclusion, combination therapy using different targets to improve antioxidant efficacy in the treatment of ALI/ARDS is very promising.
